# Unexpected presence of *Fagus orientalis *complex in Italy as inferred from 45,000-year-old DNA pollen samples from Venice lagoon

**DOI:** 10.1186/1471-2148-7-S2-S6

**Published:** 2007-08-16

**Authors:** Donatella Paffetti, Cristina Vettori, David Caramelli, Cristiano Vernesi, Martina Lari, Arturo Paganelli, Ladislav Paule, Raffaello Giannini

**Affiliations:** 1 Department of Environmental and Forestry Technologies and Sciences, University of Florence, Via San Bonaventura 13, 50145 Florence, Italy; 2 Plant Genetics Institute, National Research Council, Via Madonna del Piano 10, 50019, Sesto Fiorentino (FI), Italy; 3 Dipartimento di Biologia Animale e Genetica, Laboratorio di Antropologia, University of Florence, Via del Proconsolo 12, 50122 Firenze, Italy; 4 Centro Ecologia Alpina, Viote del Monte Bondone, 38040 Trento, Italy; 5 Department of Biology, University of Padova, Via U. Bassi 58/B, I-35121, Padova, Italy; 6 Faculty of Forestry, Technical University, SK-96053 Zvolen, Slovakia

## Abstract

**Background:**

Phylogeographic analyses on the Western Euroasiatic *Fagus *taxa (*F. orientalis*, *F. sylvatica*, *F. taurica *and *F. moesiaca*) is available, however, the subdivision of *Fagus *spp. is unresolved and there is no consensus on the phylogeny and on the identification (both with morphological than molecular markers) of *Fagus *Eurasiatic taxa.

For the first time molecular analyses of ancient pollen, dated at least 45,000 years ago, were used in combination with the phylogeny analysis on current species, to identify the *Fagus *spp. present during the Last Interglacial period in Italy.

In this work we aim at testing if the *trn*L-*trn*F chloroplast DNA (cpDNA) region, that has been previously proved efficient in discriminating different *Quercus *taxa, can be employed in distinguishing the *Fagus *species and in identifying the ancient pollen.

**Results:**

86 populations from 4 Western Euroasistic taxa were sampled, and sequenced for the *trn*L-*trn*F region to verify the efficiency of this cpDNA region in identifying the *Fagus *spp.. Furthermore, *Fagus crenata *(2 populations), *Fagus grandifolia *(2 populations), *Fagus japonica*, *Fagus hayatae*, *Quercus *species and *Castanea *species were analysed to better resolve the phylogenetic inference.

Our results show that this cpDNA region harbour some informative sites that allow to infer relationships among the species within the Fagaceae family. In particular, few specific and fixed mutations were able to discriminate and identify all the different *Fagus *species.

Considering a short fragment of 176 base pairs within the *trn*L intron, 2 transversions were found able in distinguishing the *F. orientalis *complex taxa (*F. orientalis*, *F. taurica *and *F. moesiaca*) from the remaining *Fagus *spp. (*F. sylvatica*, *F. japonica*, *F. hayataea*, *F. crenata *and *F. grandifolia*). This permits to analyse this fragment also in ancient samples, where DNA is usually highly degraded.

The sequences data indicate that the DNA recovered from ancient pollen belongs to the *F. orientalis *complex since it displays the informative sites characteristic of this complex.

**Conclusion:**

The ancient DNA sequences demonstrate for the first time that, in contrast to current knowledge based on palynological and macrofossil data, the *F. orientalis *complex was already present during the Tyrrhenian period in what is now the Venice lagoon (Italy).

This is a new and important insight considering that nowadays West Europe is not the natural area of *Fagus orientalis *complex, and up to now nobody has hypothesized the presence during the Last Interglacial period of *F. orientalis *complex in Italy.

## Background

Fossil analogs of modern Fagaceae family are well represented in the Northern Hemisphere, indicating long-term presence and differential patterns of species diversification throughout the Tertiary and Quaternary period [1-3].

In Italy during the Tyrrhenian, the last interglacial period which started around 130,000 years ago, palaeontological and palynological data support the existence in the Veneto-Po Plain of a mixed forest of Colchic type dominated by fir-trees (including *Abies nordmanniana *(Steven) Spach) and pine-trees, together with the broad-leaved trees *Zelkova *Spach, *Quercus *L., *Ulmus *L., *Tilia *L., *Carpinus *L., *Corylus *L., *Fagus *L., *Betula *L. and *Castanea *Miller [4-6].

Although a complete agreement has not been reached, most authors indicate that the Fagaceae family includes nine genera: *Fagus *L., *Castanea *L., *Castanopsis *Spach., *Chrysolepis *Hjelmquist, *Colombobalanus *(Lozano, Hdz-C. & Henao) Nixon & Crepet, *Formanodendron *(Camus) Nixon & Crepet, *Lithocarpus *Bl., *Quercus *L., and *Trigonobalanus *Forman [7]. Most genera are in temperate forests of the Northern Hemisphere, and they show a hot-spot of diversity in tropical zones of the Southern-East Asia [7].

The Fagaceae family has been extensively studied at the genetic [8,9], biogeographic and phylogeographic [10-19] level. Thanks to macrofossils richness several taxonomic and macroevolutionary studies have been performed as well [20-27]. Despite this, the taxonomic subdivision in the Fagaceae family is still controversial, due to the small number of prominent characteristics of the fruit and floral [28-30].

The traditional classification of beech in Western Eurasia recognizes one species, *F. sylvatica *with two subspecies *F. s. sylvatica *and *F. s. orientalis*, and two intermediary types, *F. moesiaca *and *F. taurica *[31,32].

*Fagus sylvatica *L. is distributed in western, central and southern Europe with marginal and isolated occurrences in England, and Scandinavia. The natural range of *F. orientalis *is Asia Minor, the Amanus Mts. (Syria), the Elburz Mts. (Iran), and Caucasus. Isolated occurrences of this taxa outside the natural range were found in eastern Serbia, in Macedonia, in Banate, in Moldova, in Dobrudja, and in central Bulgaria. Contact zones between the natural ranges of both taxa are reported in northern Greece and Bulgaria [33]. Authors disagree about the taxonomic position of *Fagus taurica *Popl. (Crimea) and *Fagus moesiaca *Czecz. (Balkan). *F. taurica *is considered by some authors as the intermediate form between *F. sylvatica *and *F. orientalis*, and by others as an independent species. Similar doubts regard the taxonomy of *F. moesiaca *considered an independent species or, most frequently, the subspecies of *F. sylvatica *[33]. The description of these taxa has been mainly based on the morphological traits of leaves [33].

Standing the difficulties in discriminating closely related taxa and the overlapping of their natural range, the molecular data by Manos and Stanford [34] provided the most reliable phylogenetic reconstruction of Fagaceae family. However, they presented limited data from European taxa, which have been traditionally included in various morphologically distinct groups. Also Denk and colleagues [32,35] tried to combine morphological and molecular data to resolve the controversy within the western Eurasiatic taxa without succeeding.

From these statements it is clear that is still controversial whether the two taxa (*F. sylvatica *and *F. orientalis*) are really separated, and whether the intermediates are two different taxonomic units (*F. taurica *and *F. moesiaca*).

Analysing populations of the Western Eurasiatic taxa *F. sylvatica *(38 populations), *F. moesiaca *(6 populations), *F. taurica *(5 populations) and *F. orientalis *(31 populations), using the *trn*L-*trn*F chloroplast DNA (cpDNA) sequence (composed: *trn*L intron, second *trn*L exon, and partial sequence of the *trn*L-*trn*F intergenic spacer), that has been previously proved efficient in discriminating *Quercus *entities [36], we aim at: (i) inferring relationships among the species within the Fagaceae family; (ii) identifying the Western Eurasiatic taxa; (iii) combining, for the first time, phylogeny analyses on present *Fagus *spp. with molecular analyses of pollen from Italian fossil specimens as old as about 45,000 yrs before present.

In addition *F. crenata *(2 populations), *F. grandifolia *(2 populations) *F. japonica *[GenBank: AB046521], *F. hayatae *[GenBank: AB046522], *Quercus *spp. and *Castanea *spp. were analysed to better resolve the phylogeny inference (see Additional file 1).

## Results and discussion

### Identification of Fagaceae family and species

The phylogenetic relationship among and within *Quercus*, *Castanea *and *Fagus *genera belonging to Fagaceae family have been inferred analysing the *trn*L-*trn*F cpDNA region in several species and in several individuals (see Additional file 1).

The entire *trn*L-*trn*F cpDNA ranged from 797 bp in *C. crenata *Sieb. to 655 bp in *F. crenata *Blume. The multiple alignment of the *trn*L-*trn*F region among the different species resulted in a sequence 889 bp long; the *trn*L intron had a length of 505 bp, the second *trn*L exon had a length of 50 bp, and the *trn*L-*trn*F partial intergenic spacer had a length of 334 bp (see Additional file 2).

The *trn*L intron belonging to intron I group is interesting for RNA secondary structure that permits to conserve active form for "autosplicing". These introns have different nucleotide primary structure, but the same secondary structure is conserved from cyanobacteria, to algae, and to plant. The conserved nucleotide regions are short four sequences named P, Q, R, and S [37]. These sequences are always present in the same order within intron, but the distance among contiguous elements range from few to hundred nucleotides [37]. The nucleotide conserved sequences in *trn*L intron were identified in *Quercus *genus by Paffetti *et al *[36]: P (TTCAGAGAAAC), Q (AATCCTGAGC), R (GTGCAGAGACTCAA) and S (AAGATAGAGTCC). In the present work the same sequences have been found in *Castanea *and *Fagus *genera.

The intron and exon of *trn*L show few nucleotides changes within Fagaceae family, particularly among the *Castanea *and *Quercus *genera according to the low rate of chloroplast DNA variation within this family, as previously shown for other cpDNA regions by several authors [7,38-40]. The *Fagus *genus is divergent from the other genera due to an insertion of 41 nucleotides within the intron region (see Additional file 2).

**Figure 1:**
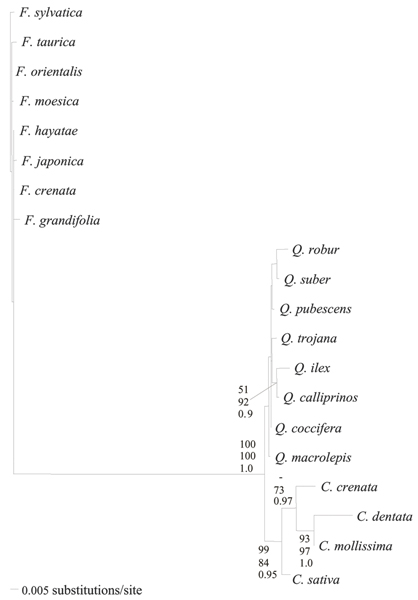
**Phylogenetic analysis**. Maximum Likelihood phylogenetic tree of present time Fagaceae family based on *trn*L-*trn*F cpDNA sequences.

The Kimura-81 model [41] with a proportion of invariant sites (0.61) and a rate variation among sites (α value of the γ distribution equal to 0.74) was selected as best-fit model of sequence evolution and then employed in Maximum Likelihood (ML) and Bayesian analyses (BI). Since trees recovered by the three different methods, Maximum Parsimony (MP), ML and BI, substantially display the same topology, we show only the ML tree (Figure 1).

The different *Fagus *species were grouped in the same unresolved cluster, providing no information about their phylogenetic relationships. High bootstrap and Bayesian posterior probability values are present only in the part of the tree involving the separation between *Castanea *and *Quercus *genera and inside the cluster of *Castanea *species.

The phylogenetic reconstruction shows a subdivision of the Fagaceae family in *Fagoideae*, *Quercoideae *and *Castanoideae *subfamily according to traditional systematic and to data obtained by Manos and Steele [39] analysing mitochondrial *mat*K gene, but in contrast with the results obtained by Nixon and Crepet [24] with macrofossil data.

Moreover, the results confirm a recent monophyletic origin of the *Fagoideae *subfamily and common origin (sister subfamily) of the *Quercoideae *and *Castanoideae *confirming data obtained by Manos and Steele [39].

The region in the alignment ranging from position 1 to 180 can distinguish the genera (see Additional file 2). Particularly, the *Fagus *genus is distinguished from *Quercus *and *Castanea *genera by four transversion (at sites 54, 78, 166 and 178) and by one transition at site 152. Moreover, the *Castanea *genus is distinguished from *Quercus *genus by one transversion at site 159.

A sequence comparative analysis of the *trn*L-*trn*F cpDNA region has been done among all the *Fagus *spp. considered (see Additional file 1).

The entire region ranged from 660 bp in *F. sylvatica *and in *F. grandifolia *Ehrh., to 655 bp in *F. crenata*. The *trnL *intron harbour some informative sites that allow to discriminate the different species inside the genus, with the intergenic region showing a higher variability at the intraspecific level. These data confirmed the results of previous studies on the genus *Quercus*, highlighting the usefulness of this sequence for taxa identification [36].

These are the most informative sites: (i) a 41 bp insertion (between positions 388 and 428) in the intron, and a 190 bp deletion (between positions 618 and 807) in the intergenic region characterise the genus *Fagus*; (ii) two transversions (site 116 and site 118) in the intron identify *F. sylvatica*; (iii) two transitions (site 116 and site 118) in the intron identify *F. grandifolia*; (iv) one transition (site 132) in the intron identifies *F. japonica *Bl.; (v) one transversion (site 439) in the intron identifies *F. moesiaca*; and (vi) two transitions (site 593 and site 596) in the intergenic identify *F. taurica *(see Additional file 2). The transversion (A/C) at site 140 is particularly interesting as it discriminates the western Eurasiatic taxa from the others. It is to notice that all the European taxa have an A, and the Asiatic species have a C. As also the other genus *Quercus *and genus *Castanea *have a C in the site 140, this is indicating that this base was present in the common ancestor of the genus *Fagaceae*, and that its mutation to A occurred in European *Fagus *more recently. Therefore, the Asiatic species are more ancient than the European ones. Moreover, *F. orientalis *seems to be originated early from the other European groups, as it presents an identical sequence to the Asiatic ones except for the site 140.

It is possible to distinguish the *F. grandifolia *species from Asiatic and western Eurasiatic taxa, and the Asiatic species from western Eurasiatic taxa; among the latter *F. orientalis*, *F. moesiaca*, *F. taurica *and *F. sylvatica *can be identified, and *F. orientalis *can be regarded as the ancestral species in European beech.

In comparison to previous works, the region analysed in this study is able to discriminate the different *Fagus *species as some mutation are fixed and specific for each *Fagus *species. Nevertheless, we also find that within *Fagus *spp. the differences are very few suggesting that speciation process within this group might be still in progress [32,35].

### Identification of ancient DNA

It is well known that DNA degradation processes pose serious limits to the length of the fragments that can be retrieved from ancient specimens [42]: only stretches of DNA less than 200 bp can be confidently analysed. Therefore, considering the age of our sample, we decided to amplify from ancient samples the *trn*L-*trn*F region ranging from site 37 to site 215, namely the region that proved useful to distinguish among the different genera (*Quercus*, *Castanea *and *Fagus*) and also among the main taxa (*F. sylvatica, F. orientalis, F. japonica, F. grandifolia*) inside the *Fagus *genus.

This region was first amplified by real-time PCR (qPCR) to estimate the copy number of cpDNA in ancient sample for the assessment of its authenticity.

A post-amplification melting curve analysis was carried out to verify that artefactual products (primer dimers, non-specific products) were not present within each reaction. A unique melting curve characteristic of our DNA fragment (different dissociation curves are obtained if different size and base composition fragments are present, as also showed by Pruvost and Geigl [43]) was obtained indicating that the reaction was problem free.

We determined that the initial amount of genuine chloroplast molecules in the fossil sample is about 59,900 molecules/μl.

One pollen grain contains about 10,000 cpDNA molecules, and five *Fagus *pollen grains are present in 1 μl of our sample (Paganelli, personal communication), so the estimated copy number is of 50,000 genomes/μl; a value very similar to the estimation made by qPCR. Therefore, we can say that our result is reliable enough for a study based on ancient DNA amplification [42].

**Figure 2:**
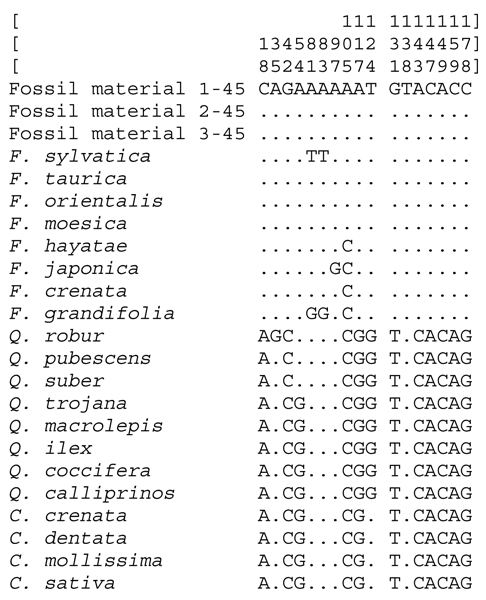
**Informative sites**. Variable informative sites among Fagaceae species analysed and fossil material.

The sequences of 176 bp obtained from fossil material, dated older than 45,000 years before present (BP) and collected near Bocca di Malamocco (Venice, Italy) (see Material and Method), belong to *Fagus *genus, since they display the informative transversions at sites 18 and 42. All the 45 clones analysed showed the same nucleotide sequences. The mutations present in these sequences are characteristic of the Western Eurasiatic group due to the presence of the transversion at site 105 position. Moreover, these sequences do not belong to *F. sylvatica *since they show transversions (A/T) at both sites 81 and 83 (Figure 2).

Therefore, the results show that *F. orientalis *complex (*F. orientalis*, *F. taurica*, *F. moesiaca*) and not *F. sylvatica *was present in Val Padana (Italy) 45,000 years BP. These data confirm the palynological data obtained by Calderoni and colleagues [44], who was not able to discriminate among the *Fagus *spp. for the limitation of the methods. Therefore, our molecular data add new crucial information to the palynological data.

### Euroasiatic *Fagus *taxa and ancient pollen grouping

**Figure 3:**
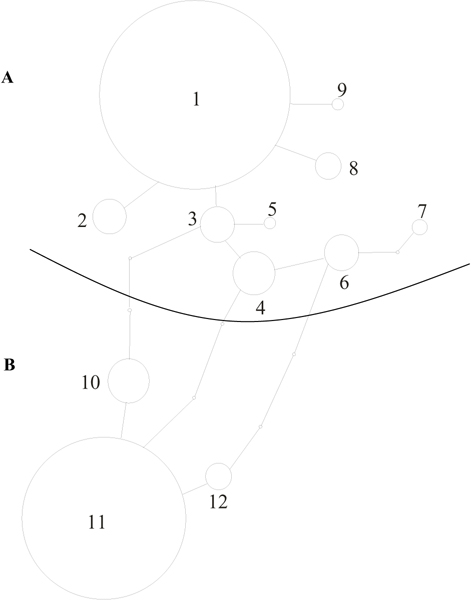
**Haplotype network**. Haplotype network for the chloroplast DNA haplotypes of *Fagus *spp. and of fossil material. Each line in the network represents a single mutation change. An haplotype is represented by a circle, and each haplotype is identify by a number. Empty circles indicate intermediate haplotypes that are not present in the sample but are necessary to link all observed haplotypes to the network.

The same 37–215 cpDNA region was used to construct a statistical parsimony network containing both ancient and modern samples of *F. sylvatica*, *F. moesiaca*, *F. taurica *and *F. orientalis *(Figure 3). Only 12 different haplotypes were recovered out of the 81 specimens analysed (80 modern plus the ancient specimen) (see Additional file 3).

The network clearly shows that two major groups, separated by at least three mutational steps, can be identified. The cluster A, comprising haplotypes from 1 to 9, is represented by the *F. orientalis, F. moesiaca*, *F. taurica *samples, plus the ancient specimens and some *F. sylvatica *samples. The cluster B, with haplotypes from 10 to 12, is formed by samples referred only to *F. sylvatica *taxa.

It is evident that the cluster B is homogeneous confirming previously data on *F. sylvatica *in western Europe [32].

The heterogeneity of cluster A is due to the presence of a mix of taxa and it can be explained, at a first attempt, by the length of the sequence used in the construction of the network, which does not contain the informative sites necessary to distinguish *F. taurica *and *F. moesiaca *from *F. orientalis*.

Another source of apparent incongruence is the presence, in this cluster A, of some populations, morphologically described as *F. sylvatica*. In effect, the sequence analysis showed that these populations have the typical sequence of the *F. orientalis*.

Nonetheless, some authors already pointed out that some central-south Italian populations look like the Balkans peninsula ones [9,19]. This seems to be the result of an ancient connection, during the Pliocene, between the Italian and Balkan peninsulas, as confirmed by previous isoenzyme analyses. This connection might have favoured gene flow and genetic admixture between the gene pool originated from the two peninsulas [9].

Considering that fossil material analysed in this study resulted belonging to the *F. orientalis *complex, another hypothesis can be put forward: these taxa were present in the Po valley 45,000 years BP. Therefore, we can hypothesize that during the interglacial period the *F. orientalis *complex was the dominant forest vegetation in Italian peninsula, and that the actual *F. sylvatica *populations (located at Foresta Umbra, Monte Taburno, Laghi di Monticchio, Monte Pecoraro, Monte Basilicò) can be considered remains of ancient populations.

Previous studies already showed that these populations are divergent from other *F. sylvatica *populations. In fact, the presence of unique haplotypes in central-southern populations was previously demonstrated [19].

Magri [45] suggested that beech populations might have survived during the last glacial period at different locations in the Italian peninsula, so that no clear large-scale migration trends can be recognised in southern and central Italy. The presence of populations displaying high divergence in Central-Southern Italy may be associated to the fact that beech populations persisted in these regions since the middle Pleistocene, as also suggested by Follieri and colleagues [46]. During the Saint Germain I (about 110,000-95,000, BP), beech represented a major component of the vegetation in central Italy. Pollen data revealed that the Italian peninsula might have been an important area for the survival of beech not only during the last glaciation, but also during previous interglacial periods [45].

Our results seem to confirm these hypotheses, adding the new information that the *F. orientalis *complex was already present.

The sporadic occurrence of *F. orientalis *in Greek and Romania populations reported in previous work [9,33] can explain the presence of some of these populations in the cluster A.

Accordingly to the results of the network, we subdivided our modern samples in two groups, *F. orientalis*-complex (cluster A) and *F. sylvatica *(cluster B), comprising respectively 51 and 29 samples. Using the entire cpDNA sequence of *Fagus *spp., 722 bp long, we submitted these groups to specific population genetics analyses. The genetic differentiation between *F. orientalis*-complex (cluster A) and *F. sylvatica *(cluster B) resulted very strong with a Φ_*st *_value equal to 0.67 (p < 0.001). The *F. orientalis*-complex (cluster A) is characterised by a value of allelic richness as three times as greater than that of *F. sylvatica *(cluster B) (Table 1). The greater genetic diversity within *F. orientalis*-complex (cluster A) is confirmed by another measure not strictly dependent on the number of samples being compared, such as the mean number of pairwise differences: we see that the *F. orientalis*-complex cluster has a value as twice as higher than that of *F. sylvatica*. We applied two statistical tests for neutrality, Tajima's D and Fu's Fs. Both tests gave statistically significant negative values only for the *F. orientalis*-complex (Table 1). Assuming neutrality selection at the locus under study, these results can be interpreted as a signature of a demographic expansion.

**Table 1:** Haplotype network analysis.

**Group**	**n**	**k**	**H**	**Ar (20)**	**π (× 100)**	**MPD**	**Fu's Fs**	**Tajima's D**
*F. orientalis *complex cluster A	51	28	0.92 ± 0.03	13.75	0.21 ± 0.14	1.55 ± 0.94	-9.43 p < 0.001	-1.94 p < 0.01
*F. sylvatica *cluster B	29	7	0.47 ± 0.11	4.84	0.0096 ± 0.02	0.07 ± 0.14	-1.18 ns	-1.15 ns

**Figure 4:**
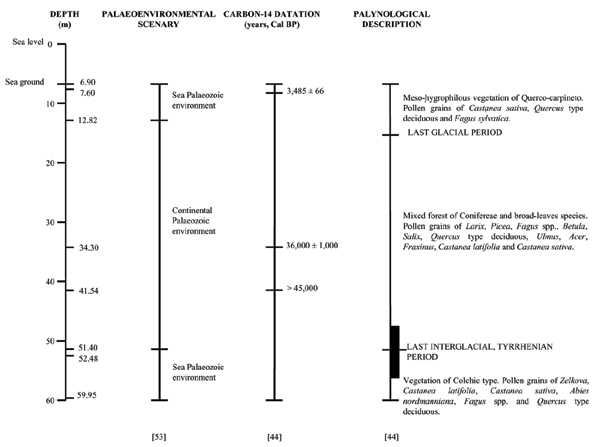
**Data describing Bocca di Malocco**. Description of the palaeo-environmental, radiocarbon and palynological data describing the Bocca di Malamocco (Venice Lagoon, Veneto, Italy) as summarized from Calderoni *et al. *[44].

Standard and molecular indexes of the *F. sylvatica *cluster A and *F. orientalis *complex as determined by the network of haplotypes (see Fig. 4 and text). Symbols used: n, sample size; k, number of different haplotypes; H, genetic diversity; Ar, allelic richness calculated with a rarefaction size of 20; π, nucleotide diversity; MPD, mean number of pairwise difference; ns, not significant.

All these results can reinforce the hypothesis according to which *F. orientalis *is the ancestral species in European beech; as also previously hypothesize by the sequence analysis. In fact, if we consider *F. sylvatica *a species derived from the ancestral *F. orientalis*, this can partially explain its reduction in genetic variability [47,48].

One general observation within Fagaceae is that sequence divergence among and within genera is fairly low. This is based on a variety of sources, including the cpDNA genes, *rbcL*, *trn*K and *matK *[34,38,39], and the ITS region [7,13]. The various noncoding regions of cpDNA sequenced in this study also reveal appreciably low levels of sequence divergence between disjoint species of most of the *Fagus *species. Estimating divergence times between species with low sequence divergence can be problematic because of low statistical power of relative rate tests and high levels of standard error [49-51].

Calculation of divergence times using the highest estimate of the rate of synonymous changes for *rbcL *(3 × 10^-10^) in Fagaceae [38], calibrated using fossil data for the minimum divergence time of *Castanea *and *Quercus *(60 million years B.P.; see Crepet [2]), provides a rough guide to estimate divergence times based on the noncoding cpDNA sequences examined here.

We adopted a coalescent-based method specifically devised for recent divergence events [52], which takes also into account the contribution of gene flow subsequent to the divergence event.

For *Fagus*, where roughly equal amounts of nucleotide variation were obtained for cpDNA noncoding regions, we calculated divergence times using the same substitution rate across the total amount of sequence. Values are derived from two different mutation rates, slow and fast which refers to the overall substitution rate (0.71 × 10^-10^) and the synonymous rate (2.36 × 10^-10^) of Frascaria *et al *[38], respectively. This suggests minimum and maximum divergence between *F. orientalis *and *F. sylvatica *of about 1.7 million and 5.74 million years BP (Table 2).

**Table 2:** Divergence times.

	**t (95% CI)**	**TMRCA**
'F. orientalis complex' vs 'F. sylvatica': slow	5,738,173 (6,230,016-2,131,321)	8,033,442
'F. orientalis complex' vs 'F. sylvatica': fast	1,700,992 (1,846,715-631,773)	2,381,291

Estimated divergence times, t, between the group denoted as 'orientalis' and 'sylvatica' as defined on the basis of genetic information (see text and Figure 3). TMRCA is the estimated time to the most recent common ancestor of the two groups. Values are derived from two different mutation rates, slow and fast which refers to the overall substitution rate (0.71 × 10^-10^) and the synonymous rate (2.36 × 10^-10^) of Frascaria *et al. *[38], respectively.

Even using the slowest mutation rate, it turns out that the two species diverged recently. The method we adopted allowed us to exclude that the relative low amount of divergence can be attributed to an older event of splitting followed by moderate gene flow.

## Conclusion

We can conclude that at the end of Tertiary period the ancestral *F. orientalis *complex was present in Italy, and that only at the beginning of the Pliocene *F. sylvatica *became differentiated from *F. orientalis*, slowly turning to a predominant role among the *Fagus *species in West Europe.

The analysis of DNA from samples dated before the Last Glacial Maximum, allowed us to provide a direct demonstration that this event dramatically affected the distribution of forest trees such as the beech.

To better understand how and why the *F. sylvatica *became predominant in comparison to *F. orientalis *in western Europe more extensive molecular study on fossils remains from different geographic area and from different ages is required, combining the results with palynological and paleoclimatic data.

## Methods

### Samples

#### Plant material

The number, distribution, source, and the GenBank accession numbers of *trn*L-*trn*F cpDNA region of individuals analysed are reported in Supplementary material (see Additional file 1).

Considering that the genetic variability and biogeography of these taxa, have been extensively studied [9-19], and that our purpose was to analyse the phylogenetic inference within this European group, we prefer to analyse as much populations as possible for each species in the attempt to adequately cover their wide natural range.

#### Fossil material

The fossil material was collected from sediments of the deep levels ranging from 48.97 m to 50.79 m of a drill, 59.95 m long (Figure 4). It was pulled up from the sea ground, located at 6.90 m below the sea level, near Bocca di Malamocco (lat. 45° 20' 00" N, long. 12° 19' 36" E), in a position corresponding to one of the three natural passages connecting the Venice Lagoon to Adriatic Sea [53]. Sediments were previously submitted to three kinds of observations by Calderoni *et al *[44]: palaeo-environmental analysis, radiocarbon dating, and palynological analysis to obtain information about vegetation and climate evolution in the investigated area. Palynological analysis was carried out on 49 levels, mainly including clay with peat and sand layers (Figure 4). The most conserved sediments, from the palynological point of view, were those consisting of peat and clay layers [44]. The extraction of "sporomorphs" were only performed from thirty-two samples according to the method described by Bertolani-Marchetti [54].

### Ancient DNA analysis

#### Authentication methods

DNA is generally present only in small amounts in ancient samples, and it is often damaged. Therefore, extreme precautions are needed to minimize the risk of amplifying and typing modern contaminating DNA molecules. To maximize the probability of extracting and sequencing authentic DNA from the ancient samples, the strictest available standards were followed throughout this study.

We have applied the following criteria to authenticate any amplified DNA sequences recovered from fossil samples [55,56].

Physically isolated work area. Fossil suspension and PCR were done in a working area sterilized by UV radiation and exclusively dedicated for this analysis. The investigators of the study wore body protection, gloves and face mask throughout the entire pre-PCR and PCR work.

1. Negative controls. PCR controls produced negative results.

2. Reproducibility. All results were identical in two independent laboratories.

3. Phylogenetic consistency. Amplified sequences showed unambiguous affinities with other Fagaceae sequences in a phylogenetic analysis, and therefore are phylogenetically consistent with their supposed Fagaceae origin.

4. Cloning. In total 57 clones were analysed and only 45 showed the expected fragment amplified. Therefore, 12 of 57 clones were excluded from the analysis as carrying partial or ambiguous fragments.

5. All results were identical in two independent extractions and two independent amplifications using four different overlapping primer pairs.

6. To explore if the number of templates was large enough (>1000) to obtain reproducible results, a quantitative Real Time PCR experiment was performed on the 176 bp fragment of the *trn*L-*trn*F chloroplast DNA (cpDNA) region.

All work was performed in isolated areas of Molecular Genetic Laboratory in Sesto Fiorentino (IGV-CNR) and in Molecular Anthropology Laboratory in Florence (DBAG-UNIFI) where modern pollen DNA analysis have never been performed.

### In Molecular Genetic Laboratory (IGV-CNR, Sesto Fiorentino)

#### Preparation of fossil samples for amplification

The working area, the disposable gloves, the automatic pipettes and all other material used for PCR were sterilised by exposition for 12 h to UV radiation (Atlas Germicidal Lamp of 30 W) with an intensity maximum of λ 254 nm at a distance of 67 cm from the working surface [57]. In addition, the laboratory is positively pressured to avoid the contamination with incoming external pollution (*e.g.*, pollen, spores, etc.).

To diminish the steps in pre-PCR amplification, and, therefore, the possibility of contamination, a modified protocol respect to the one used by Suyama *et al. *[58] and by Parducci *et al. *[59], who crushed the pollen grains in PCR tube, was used.

The fossil pollen suspension was lysed by relying on temperature shock at high temperature as follow: 14 μl of 10-fold dilutions from the fossil pollen suspension were put in PCR tubes at -20°C until completely frozen, lysed at 95°C for 10 min, and then amplified by PCR.

#### Polymerase chain reactions (PCR) conditions for ancient pollen suspension

Amplifications were performed in a 20 μl volume containing 10 mM Tris-HCl (pH 8.3), 50 mM KCl, 1.5 mM MgCl_2_, 0.001% (w/v) gelatin, 250 μM of each deoxynucleoside triphosphate, 1 μM of each primer, 14 μl of pollen suspension, and 1 U of *Platinum Taq *DNA polymerase (Invitrogen, CA, USA).

The 176 bp region was amplified using the following primers: 37 5' GAGCCAAATCCTATTTTC 3' – 215 5' ATGCTTTCTGTAGTTTTGAT 3'. After incubation for 60 s at 90°C and for 90 s at 95°C, the reaction mixtures were subjected to different cycles by using the following temperature profiles: 1) 95°C for 30 s, 60°C for 30 s, 72°C for 4 min, for 5 cycles; 2) 95°C for 30 s, 55°C for 30 s, 72°C for 4 min, for 5 cycles; and 3) 95°C for 30 s, 50°C for 30 s, 72°C for 4 min, for 25 cycles. Amplification products were then incubated at 72°C for 10 min. A Perkin Elmer 9700 thermocycler was used.

Amplification products (20 μl per lane) were analyzed by gel electrophoresis on 1% (w/v) agarose gel (Invitrogen, CA, USA) at 10 V/cm for 2 h in Tris-acetate-EDTA buffer containing 0.5 μg/ml (w/v) of ethidium bromide [60]. The gels were photographed and analyzed with an UVP scanner (Photo-Capt, Vilbert Coormat, France).

#### Quantitative estimation

The estimated copy number of chloroplast ancient DNA needs to be larger than the threshold value under which sporadic contamination can be excluded and allows assessment of the probability of its authenticity [43]. In this study, we quantified the 176 bp cpDNA region.

The real-time PCR was carried out in a laboratory dedicated exclusively to fossil DNA work taking the necessary precautions as above.

Real-time PCR (qPCR) amplification was performed in 50 μl using DyNAMO™ SYBR^® ^Green qPCR Kit (FINNZYMES, Finland) in Chromo 4™ Real-Time PCR Detector (MJ Research, California, USA), and 0.5 μM of the appropriate primers reported above. 2 μl of diluted 1:200 DNA (extracted from fossil suspension material in the molecular anthropology laboratory) were incubated with 25 μl master mix (2×) (hot start version of a modified *Tbr *DNA polymerase, SYBER Green I, optimized PCR buffer, 5 mM MgCl_2_, dNTP mix including dUTP) (Finnzymes, Finland) and 40 U/ml UDG.

Thermal cycling conditions were 50°C for 2 min (UNG treatment step): incubation with uracil-*N*-glycosylase (UNG) to remove the uracil residues from potentially contaminating DNA originating from previous amplifications. This step was followed by an incubation at 95°C for 15 min for UNG denaturation and to activate *Tbr *DNA polymerase. Amplification was carried out for 60 cycles at 94°C for 45 s, 53°C for 1 min and 72°C for 1 min, followed by melting curve step. Two serial 10-fold dilutions (E10 to E0) of the same fragment (external standard), previously amplified and purified, were included in each experiment to generate the standard curve. The amplification efficiency of both standard and target molecules were identical. At least two no-template controls were included for each experiment.

#### Cloning and sequencing

The amplification products were purified using the Agarose Gel DNA Extraction Kit (Boehringer-Mannheim, Germany) following manufacturer's specifications. The 176 bp fragments, after gel purification, were cloned into plasmid vectors to randomly sample single molecules (Herrmann and Hummel 1984). The pCR^®^2.1-TOPO vector (Invitrogen, CA, USA), and the TOPO™ TA Cloning^® ^kit (Invitrogen, CA, USA) were used. Sequencing of the cloned fragments was performed in both directions from independent amplification reactions using the dideoxy-chain termination method [61], the Sequenase kit (USB) and the M13 universal primers.

The GenBank accession number of the 176 bp fragment sequences is: DQ875545.

### In Molecular Anthropology Laboratory (DBAG-UNIFI, Florence)

#### DNA extraction

DNA was extracted from fossil material by means of a silica based protocol (modified from Krings *et al. *[62]; see Caramelli *et al. *[63]); we performed two independent extractions. A negative control was included in each extraction.

#### Amplification

We amplify the a 157 bp fragment of the *trn*L-*trn*F cpDNA overlapping the 176 bp region; the following primers were used: 17 5' AATTAAAAATGGGCAATCCT 3' – 176 5' AATAACGTAACGAAGTCAACC 3'. The following profile was used to amplify 2 μl of diluted 1: 200 DNA extracted from fossil material: 94°C for 10 min (*Taq *polymerase activation), followed by 50 cycles of PCR (denaturation 94°C for 45 s; annealing 53°C for 1 min; and extension 72°C for 1 min) and a final step at 72°C for 10 min. The 50 μl reaction mix contained 2 U of Ampli *Taq *Gold (Applied Biosystems), 200 μM of each dNTP, and 1 μM of each primer.

#### Cloning and sequencing

PCR products were cloned using the TOPO TA Cloning kit (Invitrogen), according to the manufacturer's instructions. Screening of white recombinant colonies was accomplished by PCR. The colonies were transferred into a 30 μl reaction mix (67 mM Tris HCl [pH 8.8], 2 mM MgCl_2_, 1 μM of each primer, 0.125 μM of each dNTP, and 0.75 U of *Taq *polymerase) containing M13 forward and reverse universal primers. After 5 min at 92°C, 30 cycles of PCR (30 s at 90°C, 1 min at 50°C, 1 min at 72°C) were performed and clones with an insert of the expected size were identified by agarose-gel electrophoresis. After purification of these PCR products with Microcon PCR devices (Amicon), a volume of 1.5 μl was cycle-sequenced, according to the BigDye Terminator kit (Applied Biosystems) supplier's instructions. The sequence was determined using an Applied BioSystems 3100 DNA sequencer.

The GenBank accession number of the 157 bp fragment sequences is: DQ875546.

### Modern DNA analysis

#### DNA extraction

Total DNA was extracted from dormant buds, stored at -20°C, using the DNeasy plant kit (QiAgen, Germany) following the manufacturer specifications.

#### DNA amplification by PCR

Amplifications were performed in a 20 μl volume containing 10 mM Tris-HCl (pH 8.3), 50 mM KCl, 1.5 mM MgCl_2_, 0.001% (w/v) gelatin, 250 μM of each deoxynucleoside triphosphate, 1 μM of each primer, 1 μl (10 ng) of total DNA, and 1 U of *Platinum Taq *DNA polymerase (Invitrogen, CA, USA). The oligonucleotides used as primers to amplify the *trn*L-*trn*F cpDNA region were the universal primers (c and d) described by Taberlet *et al *[64]. The PCR profiles are the same as above.

#### Sequencing

The amplification products were purified using the PCR DNA purification Kit (QiAgen, Germany) following supplier's instructions. Direct sequencing of amplified DNA was done in both directions from independent amplification reactions as above with the necessary modifications for direct PCR sequencing.

### Data analysis

The *trn*L-*trn*F cpDNA sequences were multiply aligned, using the CLUSTAL-X program [65]. Alignments were verified and adjusted manually.

Phylogenetic inferences based on the cpDNA sequence were conducted with maximum parsimony [65], maximum likelihood, ML [67] and Bayesian, BI [68] methods.

The best-fit model of nucleotide substitution employed in the ML and BI analyses was selected using the Akaike Information Content [69] approach as implemented in Modeltest 3.7 [70].

In the MP and ML analyses we adopted the TBR (Tree Bisection Reconnection) branch-swapping algorithm with respectively 150 and 100 random addition-sequence replicates. Robustness of the phylogenetic trees generated by MP and ML was tested by using the non-parametric bootstrap [71] with 1000 pseudoreplicates. The above analyses were performed with the software package PAUP* ver. 4.0 [72]. The BI analyses were carried out with the program MrBayes ver. 3.0b4 [73]. The Monte Carlo Markov Chain length was 3,000,000 generations with a sampling frequency of 100 generations. The log-likelihood values for sampled trees were stabilized after almost 200,000 generations; we used the last 20,000 out of the 30,000 total trees to estimate Bayesian posterior probabilities. From these trees a 50% majority-rule consensus tree was constructed with PAUP* ver. 4.0 [72].

The portion of the cpDNA (site 37 to site 215: 179 bp) amplified in the ancient sample was used to construct a statistical parsimony cladogram [74] within all the *Fagus spp *specimens. The statistical parsimony method joins all pairs of sequences that have a probability of parsimony greater than 0.95, that is the probability of having no unobserved mutations. The cladogram was constructed with the TCS ver 1.21 software [75].

Genetic differentiation was assessed by means of Φ_*st *_(using the Kimura-2parameters distance method with gamma correction), the molecular analogue of conventional F_*st *_[76]. We estimated Tajima's *D *[77] and Fu's *Fs *[78] to test for neutrality. All the above analyses, together with standard and molecular indexes calculation, were run with the software package Arlequin ver 2.000 [79].

Allelic richness was calculated following the method described in Comps *et al. *[16].

Classical methods do not usually allow to distinguish whether a certain level of diversity between species/populations results from a recent divergence accompanied by limited migration, or from a long divergence time during which migration was substantial. A method that can explicitly deal with this feature is that developed by Nielsen and Wakeley [52]: this is a Markov Chain Monte Carlo coalescent-based approach for obtaining maximum-likelihood estimates of three population parameters, θ (which is equal to 4N_eμ_, where N_e _is the effective populations size and μ the mutation rate), M (equal to 2N_e_m, where m is the migration rate between two populations), and T (equal to t/2N_e_, where t is the time since two populations diverged from the common ancestral population). Assuming no recombination and a demographic model of two populations of equal effective size that split from a panmittic ancestral population at some time, T, in the past, the method estimates the likelihood of the demographic parameters given the (i) demographic model, (ii) the data set (that is DNA sequences) and (iii) a mutational model. In our case we employed the finite-site Hasegawa, Kishino and Yano model (1985), which allows for recurrent mutations, differences in nucleotide frequencies and a transition/transversion bias. Adopting two mutation rates [38] we calculated N_e _and then we used this N_e _value to retrieve T and M. To check the convergence of the ergodic averages we ran the program twice starting from different seed numbers.

## Competing interests

The authors declare that they have no competing interests.

## Authors' contributions

DP, CV2, LP and RG were involved in the design phase. LP provided most of the *Fagus *spp. samples. AP provided the fossil samples and the palynological data information. CV2 carried out the molecular genetics studies. DP and CV2 made the sequence alignment and drafted the manuscript. DP and CV4 performed phylogenetic and statistical analysis. DC and ML made the molecular reproducibility of fossil sample. DC, CV4, LP, AP, and RG have been involved in revising the manuscript for important intellectual content. All authors read and approved the final manuscript.

## Supplementary Material

Additional File 1 Table S1. A complete list of the population analysed for each species and related origin information with sequence accession number. Click here for file

Additional File 2 Table S2. The multiple alignment of the *trn*L-*trn*F among the difference species. Click here for file

Additional File 3 Table S3. The list of different haplotypes obtained in the parsimony network. Click here for file
