# Supplementary material for: Unexpected presence of Fagus orientalis complex in Italy as inferred from 45,000-year-old DNA pollen samples from Venice lagoon
Source: BMC Evol Biol. 2007 Aug 16;7(Suppl 2):S6. doi: 10.1186/1471-2148-7-S2-S6 (PMC1963477; doi:10.1186/1471-2148-7-S2-S6)
Supplement: Additional File 1 — Table S1. A complete list of the population analysed for each species and related origin information with sequence accession number. [file 1471-2148-7-S2-S6-S1.pdf]

Table S1. Species of Fagaceae family used in the phylogenetic analyses. The taxonomic subdivision has been done following Tutin [31].

Taxa	Individuals N°	Origin	Coordinates	GenBank Accession N°	Source
**Subfamily *Fagoideae***					
Fagus sylvatica L.	1	Annunziata (Sicilia, Italy)	37°53’ N 14°57’ E	DQ875475	This work
	1	Laghi di Monticchio (Basilicata, Italy)	40°56’ N 15°39’ E	DQ875490	“
	1	Foresta Umbra (Puglia, Italy)	41°53’ N 16°10’ E	DQ875473	“
	1	Baia Mare (Romania)	47°50’ N 23°35’ E	DQ875468	“
	1	Gullmarsberg (Sweden)	58°22’ N 11°39’ E	DQ875477	“
	1	Westfield (Scotland)	57°40’ N 03°25’ W	DQ875500	“
	1	Lowther E. (England)	54°37’ N 02°44’ W	DQ875482	“
	1	Alsted (Denmark)	55°24’ N 11°39’ E	DQ875467	“
	1	Medingen (Germany)	53°05’ N 10°57’ E	DQ875484	“
	1	Tomaszow Lubelski (Poland)	50°37’ N 23°23’ E	DQ825497	“
	1	Tatra National Park (Slovakia)	49°16’ N 20°10’ E	DQ875495	“
	1	Tharandt (Germany)	48°83’ N 10°25’ E	DQ875496	“
	1	Slovany (Slovakia)	48°59’ N 18°44’ E	DQ875492	“
	1	Horná Súča (Slovakia)	48°56’ N 18°00’ E	DQ875478	“
	1	Zamutov (Slovakia)	48°52’ N 21°34’ E	DQ875501	“
	1	Vihorlat (Slovakia)	48°50’ N 22°02’ E	DQ875499	“
	1	Zvolen (Slovakia)	48°40’ N 19°02’ E	DQ875502	“
	1	Pol’ana (Slovakia)	48°37’ N 19°28’ E	DQ875487	“
	1	Sitno (Slovakia)	48°24’ N 18°50’ E	DQ875491	“
	1	Lozorno (Slovakia)	48°20’ N 17°08’ E	DQ875483	“
	1	Beius Bihor (Romania)	46°68’ N 22°26’ E	DQ875469	“
	1	Des Collettes (France)	46°18’ N 02°95’ W	DQ875471	“
	1	Limitaciones (Spain)	42°81’ N 02°25’ W	DQ875481	“
	1	Kladská (Czech Republic)	50°05’ N 14°26’ E	DQ875479	“
	1	Glarus (Switzerland)	47°30’ N 09°40’ E	DQ875474	“
	1	Nevrokopi (Greece)	41°11’ N 23°57’ E	DQ875486	“
	1	Pylion (Greece)	39°35’ N 22°14’ E	DQ875488	“
	1	Passo San Boldo (Veneto, Italy)	46°01’ N 12°13’ E	AF133654	[36]
	1	Monte Soro Nebrodi (Sicilia, Italy)	37°50’ N 14°43’ E	DQ875485	This work
	1	Prati di Tivo (Abruzzo, Italy)	42°31’ N 13°32’ E	DQ875476	“
	1	Pian di Novello (Toscano, Italy)	44°07’ N 10°42’ E	DQ875466	“
	1	Bosco della Martese (Abruzzo, Italy)	42°41’ N 13°30’ E	DQ875470	“
	1	Retezat (Simeria, Romania)	45°25’ N 22°50’ E	DQ875498	“
	1	Gramatikovo (Bulgaria)	42°40’ N 26°80’ E	DQ875475	“
	1	Monte Taburno (Campania, Italy)	41°04’ N 14°38’ E	DQ875494	“
	1	Monte Basilicò (Calabria, Italy)	38°09’ N 15°53’ E	DQ875489	“
	1	Lailias (Greece)	36°54’ N 21°42’ E	DQ875480	“
	1	Monte Pecoraro Sdirrocco (Calabria, Italy)	38°35’ N 16°20’ E	DQ875493	“

Fagus orientalis L.	1	Sukhansk (Cherek river, Russia)	43°21’ N 43°48’ E	DQ875517	This work
	1	Helen Gelend Caucaso (Russia)	44°35’ N 38° 11’ E	DQ875512	“
	1	Digora (Northern Osetia, Russia)	43°10’ N 44° 09’ E	DQ875509	“
	1	Devrek (Turkey)	41°10’ N 32°16’ E	DQ875508	“
	1	Eregli (Turkey)	37°03’ N 34°17’ E	DQ875511	“
	1	Vanadzor (Armenia)	40°48’ N 44°30’ E	DQ875518	“
	1	Vanadzor 3 (Armenia)	40°48’ N 44°30’ E	DQ875505	“
	1	Sochi (West Caucasus, Russia)	43°38’ N 39°42’ E	DQ875522	“
	1	Zorkun (Ammanus, Turkey)	36°58’ N 36°24’ E	DQ875532	“
	1	Neka (Iran)	36°22’ N 53°33’ E	DQ875529	“
	1	Shilda 1 (Georgia)	42°06’ N 45°50’ E	DQ875526	“
	1	Bakuriani (Georgia)	41°44’ N 43°32’ E	DQ875527	“
	1	Nichbisi (Georgia)	41°50’ N 44°33’ E	DQ875528	“
	1	Düzce (Turkey)	45°28’ N 34°07’ E	DQ875510	“
	1	Dörtyöl (Ammanus, Turkey)	36°47’ N 36°36’ E	DQ875531	“
	1	Batumi / Keda (Georgia)	41°40’ N 41°55’ E	DQ875523	“
	1	Akkus (Turkey)	40°50’ N 37°05’ E	DQ875503	“
	1	Inegoel (Turkey)	39°53’ N 29°36’ E	DQ875513	“
	1	Karabük (Turkey)	41°16’ N 32°32’ E	DQ875515	“
	1	Arboretum of Vallombrosa (Italy)	43°44’ N 11°32’ E	DQ875516	
	1	Sovetskoe (Cherek river, Russia)	43°21’ N 43°48’ E	DQ875507	This work
	1	Catalca (Turkey)	41°28’ N 28°21’ E	DQ875506	“
	1	Izmit (Turkey)	40°34’ N 29°57’ E	DQ875514	“
	1	Asalem 1900 (Iran)	37°38’ N 48°46’ E	DQ875524	“
	1	Gorgan 1400 (Iran)	36°41' N 54°05' E	DQ875519	“
	1	Kheirood 2000 (Iran)	36°28' N 51°40' E	DQ875520	“
	1	Vanadzor 2 (Armenia)	40°48’ N 44°30’ E	DQ875504	“
	1	Vanadzor 1 (Armenia)	40°48’ N 44°30’ E	AF533691	“
	1	Neka (Iran)	36°22’ N 53°33’ E	DQ875525	“
	1	Kondolovo (Bulgaria)	41°30' N 23°30' E	DQ875521	“
	1	Düzic Dumali Dag (Ammanus, Turkey)	37°16’ N 36°33’ E	DQ875530	“
Fagus taurica Popl.	1	Sokolinoe (Crimea)	44°31’ N 33°57’ E	DQ875535	“
	1	Crimean State Reserve (Crimea)	44°42’ N 34°19’ E	DQ875533	“
	1	Staryi Krym (Agarmysh, Crimea)	45°02’ N 35°01’ E	DQ875536	“
	1	Sokolinoe (Crimea)	44°30’ N 33°57’ E	AF533690	“
	1	Crimean State Reserve (Crimea)	44°39’ N 34°15’ E	DQ875534	“
Fagus moesica Czecz	1	Petrich (Bulgaria)	41°24' N 23°13' E	AF533692	“
	1	Etropole (Bulgaria)	42°50' N 24°00' E	DQ875537	“
	1	Devin (Bulgaria)	41°50' N 23°00' E	DQ875539	“
	1	Vitosha (Bulgaria)	41°55' N 24°24' E	DQ875540	“
	1	Borovetz (Bulgaria)	42°30' N 23°25' E	DQ875541	“
	1	Struma (Bulgaria)	42°24' N 23°50' E	DQ875538	“
Fagus japonica Bl.	1			AB046521	GenBank
Fagus hayatae Pablin	1			AB046522	GenBank

Fagus crenata Blume	1	Japan	DQ875542	This work
	1	Korea	AF533693	"
Fagus grandifolia Ehrh.	1	Quebec (Canada)	DQ875543	"
	1	North Carolina (USA)	DQ875544	"
**Subfamily *Quercoideae***				
Quercus robur L.	10	Münden-Low Saxony (Germany)	AF268937	[36]
Quercus pubescens Willd.	10	Radda in Chianti (Tuscany, Italy)	AF133652	"
Quercus suber L.	10	Tempio Pausania (Sardinia, Italy)	AF268938	"
Quercus trojana Webb	10	Cassano Murge (Apulia, Italy)	AF133648	"
Quercus macrolepis Kotschy	10	Tricase (Apulia, Italy)	AF133647	"
Quercus ilex L.	10	Tempio Pausania (Sardinia, Italy)	AF268939	"
Quercus coccifera L.	10	Avignon (France)	AF133650	"
Quercus calliprinos Webb	10	Haifa (Israel)	AF133649	"
**Subfamily *Castanoideae***				
Castanea crenata Sieb	2	Arboretum of Vallombrosa (Tuscany, Italy)	AF344181	This work
	1	Orchard (Beijing Province, China)	AF344181	"
Castanea dentata Borkh.	5	Experimental plot of Verbania (Lombardia, Italy)	AF344180	"
Castanea mollissima Bl.	3	Arboretum of Vallombrosa (Tuscany, Italy)	AF344182	"
Castanea sativa Mill.	1	Marradi (Tuscany, Italy)	AF133653	"
			AF344183	"
	2	Caprese Michelangelo (Tuscany, Italy)	AF133653	"
			AF344183	"
