# Supplementary material for: Unexpected presence of Fagus orientalis complex in Italy as inferred from 45,000-year-old DNA pollen samples from Venice lagoon
Source: BMC Evol Biol. 2007 Aug 16;7(Suppl 2):S6. doi: 10.1186/1471-2148-7-S2-S6 (PMC1963477; doi:10.1186/1471-2148-7-S2-S6)
Supplement: Additional File 2 — Table S2. The multiple alignment of the trnL-trnF among the difference species. [file 1471-2148-7-S2-S6-S2.pdf]

Table S2. Aligned DNA sequences of the *trn*L-*trn*F chloroplast region of the species reported in Table S1 (see Additional file 1).

```
trnL intron region (position 1-120)
                    1                                                                                                                   120
Ins/Del (D)                     D                                                 DDDDD                D                 DDDDDDDDDDDD
Transition (N)                                                                            N                  N                         N N
Transversion (V)                                                         V                       V                     V               V V
Informative (F)                                                          F                       F
F. sylvatica        TTCAGAGAAACCCTGGAATTAAAAATGGGCAATCCTGAGCCAAATCCTATTTTCCGAAAACA-----AATAAGGGTTGAGAAG-AAAGCAAGAATAAAATAAAAAAAAAAAAGGGTTTGG
F. taurica          TTCAGAGAAACCCTGGAATTAAAAATGGGCAATCCTGAGCCAAATCCTATTTTCCGAAAACA-----AATAAGGGTTGAGAAG-AAAGCAAGAATAAAATAAAAAAAAAA---GGATAGG
F. orientalis       TTCAGAGAAACCCTGGAATTAAAAATGGGCAATCCTGAGCCAAATCCTATTTTCCGAAAACA-----AATAAGGGTTGAGAAG-AAAGCAAGAATAAAATAAAAAAAAAA---GGATAGG
F. moesiaca         TTCAGAGAAACCCTGGAATTAAAAATGGGCAATCCTGAGCCAAATCCTATTTTCCGAAAACA-----AATAAGGGTTGAGAAG-AAAGCAAGAATAAAATAAAAAAAAAA---GGATAGG
F. hayatae          TTCAGAGAAACCCTGGAATTAAAAATGGGCAATCCTGAGCCAAATCCTATTTTCCGAAAACA-----AATAAGGGTTGAGAAG-AAAGCAAGAATAAAATAAAAAAAAAAA--GGATAGG
F. japonica         TTCAGAGAAACCCTGGAATTAAAAATGGGCAATCCTGAGCCAAATCCTATTTTCCGAAAACA-----AATAAGGGTTGAGAAG-AAAGCAAGAATAAAATAAAAAAAAAA---GGATAGG
F. crenata          TTCAGAGAAACCCTGGAATTAAAAATGGGCAATCCTGAGCCAAATCCTATTTTCCGAAAACA-----AATAAGGGTTGAGAAG-AAAGCAAGAATAAAATAAAAAAAA-----GGATAGG
F. grandifolia      TTCAGAGAAACCCTGGAATTAAAAATGGGCAATCCTGAGCCAAATCCTATTTTCCGAAAACA-----AATAAGGGTTGAGAAG-AAAGCAAGAATAAAATAAAAAAAAAAAAGGGGTGGG
Q. robur            TTCAGAGAAACCCTGGAATTAAAAATGGGCAATCCTGAGCCAAATCCTATTTTACGAAAACA-----AATGAGGGTTCAGAAG-AAAGCAAGAATAAAAAAA-----------GGATAGG
Q. pubescens        TTCAGAGAAACCCTGGAATTAAAAATGGGCAATCCTGAGCCAAATCCTATTTTACGAAAACA-----AATAAGGGTTCAGAAG-AAAGCAAGAATAAAAAAA-----------GGATAGG
Q. suber            TTCAGAGAAACCCTGGAATTAAAAATGGGCAATCCTGAGCCAAATCCTATTTTACGAAAACA-----AATAAGGGTTCAGAAG-AAAGCAAGAATAAAAAAA-----------GGATAGG
Q. trojana          TTCAGAGAAACCCTGGAATTAAAAATGGGCAATCCTGAGCCAAATCCTATTTTACGAAAACAAAACAAATAAGGGTTCAGAAG-AAAGCGAGAATAAAAAA------------GGATAGG
Q. macrolepis       TTCAGAGAAACCCTGGAATTAAAAATGGGCAATCCTGAGCCAAATCCTATTTTACGAAAACAAAACAAATAAGGGTTCAGAAG-AAAGCGAGAATAAAAAA------------GGATAGG
Q. ilex             TTCAGAGAAACCCTGGAATTAAAAATGGGCAATCCTGAGCCAAATCCTATTTTACGAAAACA-----AATAAGGGTTCAGAAGCAAAGCGAGAATAAAAAAA-----------GGATAGG
Q. coccifera        TTCAGAGAAACCCTGGAATTAAAAATGGGCAATCCTGAGCCAAATCCTATTTTACGAAAACA-----AATAAGGGTTCAGAAG-AAAGCGAGAATAAAAAAA-----------GGATAGG
Q. calliprinos      TTCAGAGAAACCCTGGAATTAAAAATGGGCAATCCTGAGCCAAATCCTATTTTACGAAAACA-----AATAAGGGTTCAGAAG-AAAGCGAGAATAAAAAAA-----------GGATAGG
C. crenata          TTCAGAGAAACCCTGGAATTAAAAATGGGCAATCCTGAGCCAAATCCTATTTTACGAAAACA-----AATAAGGGTTCAGAAG-AAAGCGAGAATAAAAAAAA----------GGATAGG
C. dentata          TTCAGAGAAACC-TGGAATTAAAAATGGGCAATCCTGAGCCAAATCCTATTTTACGAAAACA-----AATAAGGGTTCAGAAG-AAAGCGAGAATAAAATAA-----------GGATAGG
C. mollissima       TTCAGAGAAACC-TGGAATTAAAAATGGGCAATCCTGAGCCAAATCCTATTTTACGAAAACA-----AATAAGGGTTCAGAAG-AAAGCGAGAATAAAAAAAA----------GGATAGG
C. sativa           TTCAGAGAAACCCTGGAATTAAAAATGGGCAATCCTGAGCCAAATCCTATTTTACGAAAACA-----AATAAGGGTTCAGAAG-AAAGCGAGAATAAAAAAAA----------GGATAGG
                    ************ **************************************** ********     *** ****** ***** ***** ********* *            ** * **

trnL intron region (position 121-240)
                    121                                                                                                                 240
Ins/Del (D)                                                                                                        DDDDDDD          D
Transition (N)                 N                   N
Transversion (V)                       V                  V      V           V   V V         V                  V
Informative (F)                                    F             F           F   F F         F                  F  FFFFFFF
F. sylvatica        TGCAGAGACTCAATGGAAGATGTTCTAACAAATGGGGTTGACTTCGTTACGTTATTAAATTCAAGTAATCCTTCTATCAAAACTACAGAAAGCATGAAGGATAAACCTATAT-ACATACG
F. taurica          TGCAGAGACTCAATGGAAGATGTTCTAACAAATGGGGTTGACTTCGTTACGTTATTAAATTCAAGTAATCCTTCTATCAAAACTACAGAAAGCATGAAGGATAAACCTATAT-ACATACG
F. orientalis       TGCAGAGACTCAATGGAAGATGTTCTAACAAATGGGGTTGACTTCGTTACGTTATTAAATTCAAGTAATCCTTCTATCAAAACTACAGAAAGCATGAAGGATAAACCTATAT-ACATACG
F. moesiaca         TGCAGAGACTCAATGGAAGATGTTCTAACAAATGGGGTTGACTTCGTTACGTTATTAAATTCAAGTAATCCTTCTATCAAAACTACAGAAAGCATGAAGGATAAACCTATAT-ACATACG
F. hayatae          TGCAGAGACTCAATGGAAGCTGTTCTAACAAATGGGGTTGACTTCGTTACGTTATTAAATTCAAGTAATCCTTCTATCAAAACTACAGAAAGCATGAAGGATAAACCTATAT-ACATACG
F. japonica         TGCAGAGACTCGATGGAAGCTGTTCTAACAAATGGGGTTGACTTCGTTACGTTATTAAATTCAAGTAATCCTTCTATCAAAACTACAGAAAGCATGAAGGATAAACCTATAT-ACATACG
F. crenata          TGCAGAGACTCAATGGAAGCTGTTCTAACAAATGGGGTTGACTTCGTTACGTTATTAAATTCAAGTAATCCTTCTATCAAAACTACAGAAAGCATGAAGGATAAACCTATAT-ACATACG
F. grandifolia      TGCAGAGACTCAATGGAAGCTGTTCTAACAAATGGGGTTGACTTCGTTACGTTATTAAATTCAAGTAATCCTTCTATCAAAACTACAGAAAGCATGAAGGATAAACCTATAT-ACATACG
Q. robur            TGCAGAGACTCAATGGAAGCTGTTCTAACAAGTGGGGTGGACTTCTTTACGTTATTACATTAACGTAATCCTTATATCAAAACTACAGAAAGGAT-------AAACCTATATTACATACG
Q. pubescens        TGCAGAGACTCAATGGAAGCTGTTCTAACAAGTGGGGTGGACTTCTTTACGTTATTACATTAACGTAATCCTTATATCAAAACTACAGAAAGGAT-------AAACCTATAT-ACATACG
Q. suber            TGCAGAGACTCAATGGAAGCTGTTCTAACAAGTGGGGTGGACTTCTTTACGTTATTACATTAACGTAATCCTTATATCAAAACTACAGAAAGGAT-------AAACCTATAT-ACATACG
Q. trojana          TGCAGAGACTCAATGGAAGCTGTTCTAACAAGTGGGGTGGACTTCTTTACGTTATTACATTAACGTAATCCTTATATCAAAACTACAGAAAGGAT-------AAACCTATAT-ACATACG
Q. macrolepis       TGCAGAGACTCAATGGAAGCTGTTCTAACAAGTGGGGTGGACTTCTTTACGTTATTACATTAACGTAATCCTTATATCAAAACTACAGAAAGGAT-------AAACCTATAT-ACATACG
Q. ilex             TGCAGAGACTCAATGGAAGCTGTTCTAACAAGTGGGGTGGACTTCTTTACGTTATTACATTAACGTAATCCTTATATCAAAACTACAGAAAGGAT-------AAACCTATAT-ACATACG
Q. coccifera        TGCAGAGACTCAATGGAAGCTGTTCTAACAAGTGGGGTGGACTTCTTTACGTTATTACATTAACGTAATCCTTATATCAAAACTACAGAAAGGAT-------AAACCTATAT-ACATACG
Q. calliprinos      TGCAGAGACTCAATGGAAGCTGTTCTAACAAGTGGGGTGGACTTCTTTACGTTATTACATTAACGTAATCCTTATATCAAAACTACAGAAAGGAT-------AAACCTATAT-ACATACG
C. crenata          TGCAGAGACTCAATGGAAGCTGTTCTAACAAGTGGGGTTGACTTCTTTACGTTATTACATTAACGTAATCCTTATATCAAAACTACAGAAAGGAT-------AAACCTATAT-ACATACG
C. dentata          TGCAGAGACTCAATGGAAGCTGTTCTAACAAGTGGGGTTGACTTCTTTACGTTATTACATTAACGTAATCCTTATATCAAAACTACAGAAAGGAT-------AAACCTATAT-ACATACG
C. mollissima       TGCAGAGACTCAATGGAAGCTGTTCTAACAAGTGGGGTTGACTTCTTTACGTTATTACATTAACGTAATCCTTATATCAAAACTACAGAAAGGAT-------AAACCTATAT-ACATACG
C. sativa           TGCAGAGACTCAATGGAAGCTGTTCTAACAAGTGGGGTTGACTTCTTTACGTTATTACATTAACGTAATCCTTATATCAAAACTACAGAAAGGAT-------AAACCTATAT-ACATACG
                    *********** ******* *********** ****** ****** *********** *** * ********* ****************** **       ********** *******
```

trnL intron region (position 241-360)

```
                    241                                                                                                                  360
Ins/Del (D)                                                                  DDDD              D  DDDDDDDDDD
Transition (N)                                                                          N
Transversion (V)                                  V          VV                         V   V    V
Informative (F)                                   F          F                                F           F
F. sylvatica        TATATGTACTGAAATCCTATCTCAAATGATTAATGACGACCGGAATCTTTATTTATTTATATTTCTATAAATAAATA--------C-CGAAAGAGTTGTTGTGAATCGATCCAAATTGAA
F. taurica          TATATGTACTGAAATCCTATCTCAAATGATTAATGACGACCGGAATCTTTATTTATTTATATTTCTATAAATAAATA--------C-CGAAAGAGTTGTTGTGAATCGATCCAAATTGAA
F. orientalis       TATATGTACTGAAATCCTATCTCAAATGATTAATGACGACCGGAATCTTTATTTATTTATATTTCTATAAATAAATA--------C-CGAAAGAGTTGTTGTGAATCGATCCAAATTGAA
F. moesiaca         TATATGTACTGAAATCCTATCTCAAATGATTAATGACGACCGGAATCTTTATTTATTTATATTTCTATAAATAAATA--------C-CGAAAGAGTTGTTGTGAATCGATCCAAATTGAA
F. hayatae          TATATGTACTGAAATCCTATCTCAAATGATTAATGACGACCGGAATCTTTATTTATTTATATTTCTATAAATAAATA--------C-CGAAAGAGTTGTTGTGAATCGATCCAAATTGAA
F. japonica         TATATGTACTGAAATCCTATCTCAAATGATTAATGACGACCGGAATCTTTATTTATTTATATTTCTATAAATAAATA--------C-CGAAAGAGTTGTTGTGAATCGATCCAAATTGAA
F. crenata          TATATGTACTGAAATCCTATCTCAAATGATTAATGACGACCGGAATCTTTATTTATTTATATTTCTATAAATAAATA--------C-CGAAAGAGTTGTTGTGAATCGATCCAAATTGAA
F. grandifolia      TATATGTACTGAAATCCTATCTCAAATGATTAATGACGACCGGAATCTTTATTTATTTATATTTCTATAAATAAATA--------C-CGAAAGAGTTGTTGTGAATCGATCCAAATTGAA
Q. robur            TATATGTACTGAAATCCTATCTCAAATGATAAATGACGACCGGAATCTTTATTTATTTATATTTCTATAAATAA-TAAATAAAAACTCGAAAGAGTTGTTGTGAATCGATCCAAATTGAA
Q. pubescens        TATATGTACTGAAATCCTATCTCAAATGATAAATGACGACCGGAATCTTTATTTATTTATATTTCTATAAATAA-TAAATAAAAA-TCGAAAGAGTTGTTGTGAATCGATCCAAATTGAA
Q. suber            TATATGTACTGAAATCCTATCTCAAATGATAAATGACGACCGGAATCTTTATTTATTTATATTTCTATAAATAA-TAAATAAAAA-TCGAAAGAGTTGTTGTGAATCGATCCAAATTGAA
Q. trojana          TATATGTACTGAAATCCTATCTCAAATGATAAATGACGACCGGAATCTTTATTTATTTATATTTCTATAAATAA-TAAATAAAAA-TCGAAAGAGTTGTTGTGAATCGATCCAAATTGAA
Q. macrolepis       TATATGTACTGAAATCCTATCTCAAATGATAAATGACGACCGGAATCTTTATTTATTTATATTTCTATAAATAA-TAAATAAAAA-TCGAAAGAGTTGTTGTGAATCGATCCAAATTGAA
Q. ilex             TATATGTACTGAAATCCTATCTCAAATGATAAATGACGACCGGAATCTTTATTTAT----ATTTCTATAAATAA-TAAATAAAAA-TCGAAAGAGTTGTTGTGAATCGATCCAAATTGAA
Q. coccifera        TATATGTACTGAAATCCTATCTCAAATGATAAATGACGACCGGAATCTTTATTTATTTATATTTCTATAAATAA-TAAATAAAAA-TCGAAAGAGTTGTTGTGAATCGATCCAAATTGAA
Q. calliprinos      TATATGTACTGAAATCCTATCTCAAATGATAAATGACGACCGGAATCTTTATTTAT----ATTTCTATAAATAA-TAAATAAAAA-TCGAAAGAGTTGTTGTGAATCGATCCAAATTGAA
C. crenata          TATATGTACTGAAATCCTATCTCAAATGATTAATGACGACCCGAATCTTTATTTATTTATATTTCTATCAATAA-TAAATAAAAA-TCGAAAGAGTTGTTGTGAATCGATCCAAATTGAA
C. dentata          TATATGTACTGAAATCCTATCTCAAATGATTAATGACGACCCCAATCTTTATTTATTTATATTTCTATGAATCA-TACATAAAA-CTCGAAAGAGTTGTTGTGACTCCATCCAAATTGAA
C. mollissima       TATATGTACTGAAATCCTATCTCAAATGATTAATGACGACCCGAATCTTTATTTATTTATATTTCTATAAATAA-TAAATAAAAA-TCGAAAGAGTTGTTGTGAATCGATCCAAATTGAA
C. sativa           TATATGTACTGAAATCCTATCTCAAATGATTAATGACGACCCGAATCTTTATTTATTTATATTTCTATAAATCA-TAAATAAAAA-TCGAAAGAGTTGTTGTGAATCGATCCAAATTGAA
                    ****************************** **********  *************    ******** *** * **         **********************************
```

trnL intron region (position 361-480)

```
                    361                                                                                                                  480
Ins/Del (D)                                DD  DDDDDDDDDDDDDDDDDDDDDDDDDDDDDDDDDDDDDDDDD
Transition (N)                                                                                                              N   N
Transversion (V)               V                                                                  V                          V      V
Informative (F)                                F FFFFFFFFFFFFFFFFFFFFFFFFFFFFFFFFFFFFFFFF
F. sylvatica        GAAAGAATCGAATATTTATTAATTCAATTATTTACTCCATCATAGTCTGATAGATCTTTTGAAGAACTAATTTATCGTACGAGAATAAAGATAGAGTCCCATTCTACATGTCAATACCGA
F. taurica          GAAAGAATCGAATATTTATTAATTCAATTATTTACTCCATCATAGTCTGATAGATCTTTTGAAGAACTAATTTATCGTACGAGAATAAAGATAGAGTCCCATTCTACATGTCAATACCGA
F. orientalis       GAAAGAATCGAATATTTATTAATTCAATTATTTACTCCATCATAGTCTGATAGATCTTTTGAAGAACTAATTTATCGTACGAGAATAAAGATAGAGTCCCATTCTACATGTCAATACCGA
F. moesiaca         GAAAGAATCGAATATTTATTAATTCAATTATTTACTCCATCATAGTCTGATAGATCTTTTGAAGAACTAATTTATCGTCCGAGAATAAAGATAGAGTCCCATTCTACATGTCAATACCGA
F. hayatae          GAAAGAATCGAATATTTATTAATTCAATTATTTACTCCATCATAGTCTGATAGATCTTTTGAAGAACTAATTTATCGTACGAGAATAAAGATAGAGTCCCATTCTACATGTCAATACCGA
F. japonica         GAAAGAATCGAATATTTATTAATTCAATTATTTACTCCATCATAGTCTGATAGATCTTTTGAAGAACTAATTTATCGTACGAGAATAAAGATAGAGTCCCATTCTACATGTCAATACCGA
F. crenata          GAAAGAATCGAATATTTATTAATTCAATTATTTACTCCATCATAGTCTGATAGATCTTTTGAAGAACTAATTTATCGTACGAGAATAAAGATAGAGTCCCATTCTACATGTCAATACCGA
F. grandifolia      GAAAGAATCGAATATTTATTAATTCAATTATTTACTCCATCATAGTCTGATAGATCTTTTGAAGAACTAATTTATCGTACGAGAATAAAGATAGAGTCCCATTCTACATGTCAATACCGA
Q. robur            GAAAGAATCGAATATTTATTAAT--AA-----------------------------------------AATTTATCGTACGAGAATAAAGATAGAGTCCCATTCCACACGTCAATACCGA
Q. pubescens        GAAAGAATCGAATATTTATTAAT--AA-----------------------------------------AATTTATCGTACGAGAATAAAGATAGAGTCCCATTCCACACGTCAATACCGA
Q. suber            GAAAGAATCGAATATTTATTAAT--AA-----------------------------------------AATTTATCGTACGAGAATAAAGATAGAGTCCCATTCCACACGTCAATACCGA
Q. trojana          GAAAGAATCGAATATTTATTAAT--AA-----------------------------------------AATTTATCGTACGAGAATAAAGATAGAGTCCCATTCCACACGTCAATACCGA
Q. macrolepis       GAAAGAATCGAATATTTATTAAT--AA-----------------------------------------AATTTATCGTACGAGAATAAAGATAGAGTCCCATTCCACACGTCAATACCGA
Q. ilex             GAAAGAATCGAATATTTATTAAT--AA-----------------------------------------AATTTATCGTACGAGAATAAAGATAGAGTCCCATTCCACACGTCAATACCGA
Q. coccifera        GAAAGAATCGAATATTTATTAAT--AA-----------------------------------------AATTTATCGTACGAGAATAAAGATAGAGTCCCATTCCACACGTCAATACCGA
Q. calliprinos      GAAAGAATCGAATATTTATTAAT--AA-----------------------------------------AATTTATCGTACGAGAATAAAGATAGAGTCCCATTCCACACGTCAATACCGA
C. crenata          GAAAGAATCGAATATTTATTAAT--AA-----------------------------------------AATTTATCGTACGAGAATAAAGATAGAGTCCCATTCCACACGTCTATACCGA
C. dentata          GAAAGAATCGAATATTTATTAAT--AA-----------------------------------------AATTTATCGTACGAGAATAAAGATAGAGTCCCATTCCCCACGTCTATACCGA
C. mollissima       GAAAGAATCGACTATTTATTAAT--AA-----------------------------------------AATTTATCGTACGAGAATAAAGATAGAGTCCCATTCCCCACGTCTATACCGA
C. sativa           GAAAGAATCGACTATTTATTAAT--AA-----------------------------------------AATTTATCGTACGAGAATAAAGATAGAGTCCCATTCCACACGTCTATACCGA
                    *********** ***********  **                                         ********** *************************  ** *** *******
```

trnL intron region (position 481-505) trnL exon (position 506-555) trnL-trnF intergenic region (position 556-600)

```
                    481                                                                                                                  600
Ins/Del (D)                            D                                                                                 D
Transition (N)                           NN                                    N                            N  N    N               N NN
Transversion (V)                      V                                      V                                                 V       V
Informative (F)                       FF                                     F F                               F    F                 F
F. sylvatica        CAAGAATGAAATTTATAGT-AAGAGGAAAATCCGTCGACTTTAGAAATCGTGAGGGTTCAAGTCCCTCTATCCCCAAAAAGGCCCGTTTGACTCCCTAATT-ATTTATCCGATCTTCTCT
F. taurica          CAAGAATGAAATTTATAGT-AAGAGGAAAATCCGTCGACTTTAGAAATCGTGAGGGTTCAAGTCCCTCTATCCCCAAAAAGGCCCGTTTGACTCCCTAATT-ATTTATCCGACCTCCTCT
F. orientalis       CAAGAATGAAATTTATAGT-AAGAGGAAAATCCGTCGACTTTAGAAATCGTGAGGGTTCAAGTCCCTCTATCCCCAAAAAGGCCCGTTTGACTCCCTAATT-ATTTATCCGATCTTCTCT
F. moesiaca         CAAGAATGAAATTTATAGT-AAGAGGAAAATCCGTCGACTTTAGAAATCGTGAGGGTTCAAGTCCCTCTATCCCCAAAAAGGCCCGTTTGACTCCCTAATT-ATTTATCCGATCTTCTCT
F. hayatae          CAAGAATGAAATTTATAGT-AAGAGGAAAATCCGTCGACTTTAGAAATCGTGAGGGTTCAAGTCCCTCTATCCCCAAAAAGGCCCGTTTGACTCCCTAATT-ATTTATCCGATCTTCTCT
F. japonica         CAAGAATGAAATTTATAGT-AAGAGGAAAATCCGTCGACTTTAGAAATCGTGAGGGTTCAAGTCCCTCTATCCCCAAAAAGGCCCGTTTGACTCCCTAATT-ATTTATCCGATCTTCTCT
F. crenata          CAAGAATGAAATTTATAGT-AAGAGGAAAATCCGTCGACTTTAGAAATCGTGAGGGTTCAAGTCCCTCTATCCCCAAAAAGGCCCGTTTGACTCCCTAATT-ATTTATCCGATCTTCTCT
F. grandifolia      CAAGAATGAAATTTATAGT-AAGAGGAAAATCCGTCGACTTTAGAAATCGTGAGGGTTCAAGTCCCTCTATCCCCAAAAAGGCCCGTTTGACTCCCTAATT-ATTTATCCGATCTTCTCT
Q. robur            CAAGAATGAAATTTATAGG-AAGAGGAAAATCCGTCGACTTTAGAAATCGTGAGGGTTCGAGTCCCTCTATCCCCAAAAAGGCCCGTTTGATTCCCCAATT-ATTTATCCGATCCGCTCT
Q. pubescens        CAAGAATGAAATTTATAGG-AAGAGGAAAATCCGTCGACTTTAGAAATCGTGAGGGTTCGAGTCCCTCTATCCCCAAAAAGGCCCGTTTGATTCCCCAATT-ATTTATCCGATCCGCTCT
Q. suber            CAAGAATGAAATTTATAGG-AAGAGGAAAATCCGTCGACTTTAGAAATCGTGAGGGTTCGAGTCCCTCTATCCCCAAAAAGGCCCGTTTGATTCCCCAATT-ATTTATCCGATCCGCTCT
Q. trojana          CAAGAATGAAATTTATAGG-AAGAGGAAAATCCGTCGACTTTAGAAATCGTGAGGGTTCGAGTCCCTCTATCCCCAAAAAGGCCCGTTTGATTCCCCAATT-ATTTATCCGATCCGCTCT
Q. macrolepis       CAAGAATGAAATTTATAGGGAAGAGGAAAATCCGTCGACTTTAGAAATCGTGAGGGTTCGAGTCCCTCTATCCCCAAAAAGGCCCGTTTGATTCCCCAATTTATTTATCCGATCCGCTCT
Q. ilex             CAAGAATGAAATTTATAGG-AAGAGGAAAATCCGTCGACTTTAGAAATCGTGAGGGTTCGAGTCCCTCTATCCCCAAAAAGGCCCGTTTGATTCCCCAATT-ATTTATCCGATCCGCTCT
Q. coccifera        CAAGAATGAAATTTATAGG-AAGAGGAAAATCCGTCGACTTTAGAAATCGTGAGGGTTCGAGTCCCTCTATCCCCAAAAAGGCCCGTTTGATTCCCCAATT-ATTTATCCGATCCGCTCT
Q. calliprinos      CAAGAATGAAATTTATAGG-AAGAGGAAAATCCGTCGACTTTAGAAATCGTGAGGGTTCGAGTCCCTCTATCCCCAAAAAGGCCCGTTTGATTCCCCAATT-ATTTATCCGATCCGCTCT
C. crenata          CAAGAATGAAATTTATAGG-AGGAGGAAAATCCGTCGACTTTAGAAATCGTGAGGGTGCGAGTCCCTCTATCCCCAAAAAGGCCCGTTCGATTCCCCAATT-ATTTAGCCGATCCGCTCT
C. dentata          CAAGAATGAAATTTATAGG-AGGAGGAAAATCCGTCGACTTTAGAAATCGTGAGGGTGCGAGTCCCTCTATCCCCAAAAAGGCCCGTTTGATTCCCCAATT-ATTTAGCCGATCCGCTCT
C. mollissima       CAAGAATGAAATTTATAGG-AGGAGGAAAATCCGTCGACTTTAGAAATCGTGAGGGTGCGAGTCCCTCTATCCCCAAAAAGGCCCGTTTGATTCCCCAATT-ATTTATCCGATCCGCTCT
C. sativa           CAAGAATGAAATTTATAGG-AGAAGGAAAATCCGTCGACTTTAGAAATCGTGAGGGTGCGAGTCCCTCTATCCCCAAAAAGGCCCGTTTGATTCCCCAATT-ATTTATCCGATCCGCTCT
                    ******************  *  ********************************** * **************************** ** **** **** ***** **** *  ****
```

trnL-trnF intergenic region (position 601-720)

```
                    601                                                                                                                  720
Ins/Del (D)                     D    DDDDDDDDDDDDDDDDDDDDDDDDDDDDDDDDDDDDDDDDDDDDDDDDDDDDDDDDDDDDDDDDDDDDDDDDDDDDDDDDDDDDDDDDDDDDDDDDDDDDDDD
Transition (N)              N N N                                     N                   N N N              N
Transversion (V)              VV    V       V                                          V                                   V VV          V V
Informative (F)             F       F
F. sylvatica        TTTCGTTTTGTTAGCGT-------------------------------------------------------------------------------------------------------
F. taurica          TTTCGTTTTGTTAGCGT-------------------------------------------------------------------------------------------------------
F. orientalis       TTTCGTTTTGTTAGCGT-------------------------------------------------------------------------------------------------------
F. moesiaca         TTTCGTTTTGTTAGCGT-------------------------------------------------------------------------------------------------------
F. hayatae          TTTCGTTTTGTTAGCGT-------------------------------------------------------------------------------------------------------
F. japonica         TTTCGTTTTGTTAGCGT-------------------------------------------------------------------------------------------------------
F. crenata          TTTCGTTTTGTTAGCGT-------------------------------------------------------------------------------------------------------
F. grandifolia      TTTCGTTTTGTTAGCGT-------------------------------------------------------------------------------------------------------
Q. robur            TTTCGTTTCGAAAGCGG----TTTCAAATTCGTTATGTTTTTCAATCATTC-AACTCTTTTAACAAATGGATC-GGGGTTGG---AAATTTTTTTTTTT--CTTATTACAATATTTGTGA
Q. pubescens        TTTCGTTTCGTAAGCGG----TTTAAAATTCGTTATGTTTTTCAATCATTCTA-CTCTTTTAACAAATGG-TCTG-A-TTGGGG----TTTTTTTT-----CTTATTACAATATTTGTGA
Q. suber            TTTCGTTTCGAAAGCGG----TTTCAAATTCGT-ATGTTTTTCAATCATTCTA-CTCTTTTAACAAATGGATCTG-A-TTGG---AAATTTTTTTT-----CTTATTACAATATTTGTGA
Q. trojana          TTTCGTTTCGAAAGCGG----TTTAAAATTCGTTATGTTTTTCAATCATTC-AACTCTTT-AACAAATGGATCTG-A-TTGG---AAATTTTTTTTT----CTTATTACAATATTTGTGA
Q. macrolepis       TTTCGTTTCGTT-GCGG----TTTAAAATTCGTTATGTTTTTCAATCATTCTA-CTCTTTTA-CAAATGGATCTG-A-TTGGGG-AAATTTTTTTTT----CTTATTACAATATTTGTGA
Q. ilex             TTTCGTTTCGGAAGCGGG----TTAAAATTCGTTATGTTTTTCAATCATTTTA--T-TTTTAACAAATGGATC-GGA-TTGG---AAATCTTTTTTTT---CTTATTACAATATTTGTGA
Q. coccifera        TTTCGTTTCGTAAGCGG----TTTAAAATTCGTTATGTTTTTCAATCATTCTA-CTCTTTTA-CAAATGGATCTGGA-TTGG---AAATTTTTTTTT----CTTATTACAATATTTGTGA
Q. calliprinos      TTTCGTTTCGTAAGCGG----TTTAAAATTCGTTATGTTTTTCAATCATTCTA-CTCTTT-AACAAATGGATCTGGA-TTGG---AAATTTTTTTTT----CTTATTACAATATTTGTGC
C. crenata          TTTCGTTTCGTTGGCGGGCGGTTTAAAATTCGTTATGTTTTTCAATCATTTTAACT-TTTTA-CAAATGGGTCTGGA-TTGGGGGAAATTTTTTTTT----CTTATTACAATATTTGTGA
C. dentata          TTTCGTTTCGTTAGCGG----TTTAAAATTCGTTATGTTTT-CAATC-TTTTAA-TCTTTTAACAAAAGGGTTTGGGG--GGGGGAAATTTTTTTTTTTTT--AAAAACAATATTTGGGA
C. mollissima       TTTCGTTTCGTTAGCGG----TTTAAAATTCGTTATGTTTT-CAATC-TTTTAA-TCTTTTA-CAAATGGGTTTGGGG--GGGGGAAATTTTTTTTTTTTTCTTATTACAATATTTGTGA
C. sativa           TTTCGTTTCGTTAGCGGG---TTTCAAATTCGTTATGTTTT-CAATCATTC-AACTCTTTTA-CAAATGGATCTG-A-TTGGGGGAAATTTTTTTTTTTTTCTTATTACAATATTTGTGA
                    ******** *   ***
```

trnL-trnF intergenic region (position 721-840)

```
                    721                                                                                                                  840
Ins/Del (D)         DDDDDDDDDDDDDDDDDDDDDDDDDDDDDDDDDDDDDDDDDDDDDDDDDDDDDDDDDDDDDDDDDDDDDDDDDDDDDDDDDDDDDDD                          DDDDDDD
Transition (N)                  N             N  N             N                                             NNN     N  N NN
Transversion (V)          V  V                                                                              V   V            V V
Informative (F)
F. sylvatica        ---------------------------------------------------------------------------------------TTTCAAATTGGAATTCGTTATGTTTTTCAATCA
F. taurica          ---------------------------------------------------------------------------------------TTTCAAATTGGAATTCGTTATGTTTTTCAATCA
F. orientalis       ---------------------------------------------------------------------------------------TTTCAAATTGGAATTCGTTATGTTTTTCAATCA
F. moesiaca         ---------------------------------------------------------------------------------------TTTCAAATTGGAATTCGTTATGTTTTTCAATCA
F. hayatae          ---------------------------------------------------------------------------------------TTTCAAATTGGAATTCGTTATGTTTTTCAATCA
F. japonica         ---------------------------------------------------------------------------------------TTTCAAATTGGAATTCGTTATGTTTTTCAATCA
F. crenata          ---------------------------------------------------------------------------------------TTTCAAATTGGAATTCGTTATGTTTTTCAATCA
F. grandifolia      ---------------------------------------------------------------------------------------TTTCAAATTGGAATTCGTTATGTTTTTCAATCA
Q. robur            TATATATGATACGCGTACAAATGAACGTCTTTGAGGAAGGTATCCCCACTTCCAATTTGAATGATTAACAATACATATCATTTCTTGTACTGTATTAAAACTTATAAAGTTTT-------
Q. pubescens        TATATATGATACACGTACAAATGAACGTCTTTGAGGAAGGTATCCCCACTTCCAATTTGAATGATTAACAATACATATCATTTCTTGTACTGTATTAAAACTTATAAAGTTTT-------
Q. suber            TATATATGATACGCGTACAAATGAACGTCTTTGAGGAAGGTATCCCCACTTCCAATTTGAATGATTAACAATACATATCATTTCTTGTACTGTATTAAAACTTATAAAGTTTT-------
Q. trojana          TATATATGATACGCGTACAAATGAACGTCTTTGAGGAAGGTATCCCCACTTCCAATTTGAATGATTAACAATACATATCATTTCTTGTACTGTATTAAAACTTATAAAGTTTT-------
Q. macrolepis       TATATATGATACGCGTACAAATGAACGTCTTTGAGGAAGGTATTCCCACTTCCAATTTGAATGATTAACAATACATATCATTTCTTGTACTGTATTAAAACTTATAAAGTTTT-------
Q. ilex             TATATATGATACGCGTACAAATGAACATCTTTGAGGAAGGTATCCCCACTTCCAATTTGAATGATTAACAATACATATCATTTCTTGTACTGTATTAAAACTTATAAAGTTTT-------
Q. coccifera        TATATATGATACGCGTACAAATGAACGTCTTTGAGGAAGGTATCCCCACTTCCAATTTGAATGATTAACAATACATATCATTTCTTGTACTGTATTAAAACTTATAAAGTTTT-------
Q. calliprinos      TATATATGATACGCGTACAAATGAACATCTTTGAGGAAGGTATCCCCACTTCCAATTTGAATGATTAACAATACATATCATTTCTTGTACTGTATTAAAACTTATAAAGTTTT-------
C. crenata          TATATATGATACGCGTACAAATGAACATCCTTGAGGAAGGTATCCCCACTTCCAATTTGAATGATTAACAATACATATCATTTCTTGTACTGTATTAAAACTTATAAAGTTTT-------
C. dentata          TATATAGGAAACGCGTACAAATGAAGATCTTTGAGGAAGGTATCCCCACTTCCAATTTGAATGATTAACAATACATATCATTTCTTGTACTGTATTAAAACTTATAAAGTTTT-------
C. mollissima       TATATATGATACGCGTACAAATGAACATCTTTGAGGAAGGTATCCCCACTTCCAATTTGAATGATTAACAATACATATCATTTCTTGTACTGTATTAAAACTTATAAAGTTTT-------
C. sativa           TATATATGATACGCGTACAAATGAACGTCTTTGAGGAAGGTATCCCCACTTCCAATTTGAATGATTAACAATACATATCATTTCTTGTACTGTATTAAAACTTATAAAGTTTT-------
                                                                                                             ***  ** *  * * *****
```

trnL-trnF intergenic region (position 841-884)

```
                    841                                           889
Ins/Del (D)         DDDDDDD     DDDDDDDDDD     DDDD        D  DDDDDD
Transition (N)                                        N
Transversion (V)                        V  V            V
Informative (F)
F. sylvatica        TTCTACTCTTTT----------ACAAAT----GGATCTG-AT------T
F. taurica          TTCTACTCTTTT----------ACAAAT----GGATCTG-AT------T
F. orientalis       TTCTACTCTTTT----------ACAAAT----GGATCTG-AT------T
F. moesiaca         TTCTACTCTTTT----------ACAAAT----GGATCTG-AT------T
F. hayatae          TTCTACTCTTTT----------ACAAAT----GGATCTG-AT------T
F. japonica         TTCTACTCTTTT----------ACAAAT----GGATCTG-AT------T
F. crenata          TTCTACTCTTTT----------ACAAAT----GGATCTG-AT------T
F. grandifolia      TTCTACTCTTTT----------ACAAAT----GGATCTG-AT------T
Q. robur            -------CTTTTTGAAGATCGAAGAAATTACAGGGTCTGGATAAAGCTT
Q. pubescens        -------CTTTTTGAAGATCGAAGAAATTACAGGGTCTGGATAAAGCTT
Q. suber            -------CTTTTTGAAGATCCAAGAAATTACAGGGTCTGGATAAAGCTT
Q. trojana          -------CTTTTTGAAGATCGAAGAAATTACAGGGTCTGGATAAAGCTT
Q. macrolepis       -------CTTTTTGAAGATCGAAGAAATTACAGGGTCTGGATAAAGCTT
Q. ilex             -------CTTTTTGAAGATCCAAGAAATTACAGGGTCTGGATAAAGCTT
Q. coccifera        -------CTTTTTGAAGATCGAAGAAATTACAGGGTCTGGATAAAGCTT
Q. calliprinos      -------CTTTTTGAAGATCCAAGAAATTACAGGGTCTGGATAAAGCTT
C. crenata          -------CTTTTTGAAGATCCAAGAAATTACAGGGTATGGATAAAGCTT
C. dentata          -------CTTTTTGAAGATCCAAGAAATTACAGGGTCTGGATAAAGCTT
C. mollissima       -------CTTTTTGAAGATCCAAGAAATTACAGGGTCTGGATAAAGCTT
C. sativa           -------CTTTTTGAAGATCGAAGAAATTACAGGGTCTGGATAAAGCTT
                           *****          * ****    ** * ** **      *
```
