# Supplementary material for: Unexpected presence of Fagus orientalis complex in Italy as inferred from 45,000-year-old DNA pollen samples from Venice lagoon
Source: BMC Evol Biol. 2007 Aug 16;7(Suppl 2):S6. doi: 10.1186/1471-2148-7-S2-S6 (PMC1963477; doi:10.1186/1471-2148-7-S2-S6)
Supplement: Additional File 3 — Table S3. The list of different haplotypes obtained in the parsimony network. [file 1471-2148-7-S2-S6-S3.pdf]

Table S3. List of different haplotypes depicted in the statistical parsimony network based on a 176 bp long fragment of the cpDNA. The absolute frequency of each haplotype is reported in the third column.

Original samples	Haplotype label	Total haplotypes (n)
F. o. Vanadzor (Armenia)<br>F. o. Vanadzor 1 (Armenia)<br>F. o. Vanadzor 2 (Armenia)<br>F. o. Vanadzor 3 (Armenia)<br>F. o. Catalca (Turkey)<br>F. o. Sovetskoe (Cherek river, Russia)<br>F. t. Crimean State Reserve (Crimea)<br>F. t. Crimean State Reserve (Crimea)<br>F. t. Sokolinoe (Crimea)<br>F. t. Sokolinoe (Crimea)<br>F. o. Devrek (Turkey)<br>F. o. Digora (Northern Osetia, Russia)<br>F. o. Düzce (Turkey)<br>F. o. Eregli (Turkey)<br>F. s. Foresta Umbra (Puglia, Italy)<br>F. s. Gramaticovo (Bulgaria)<br>F. o. Helen Gelend Caucaso (Russia)<br>F. o. Izmit (Turkey)<br>F. s. Lailias (Greece)<br>F. o. Arboretum of Vallombrosa (Italy)<br>F. o. Neka (Iran)<br>F. o. Gorgan (Iran)<br>F. o. Kheirood (Iran)<br>F. m. Petrich (Bulgaria)<br>F. m. Etropole (Bulgaria)<br>F. m. Devin (Bulgaria)<br>F. m. Vitosha (Bulgaria)<br>F. t. Staryi Krym (Agarmysh, Crimea)<br>F. o. Sukhansk (Cherek river, Russia)<br>F. s. Monte Taburno (Campania, Italy)<br>F. o. Sochi (Western Caucaso, Russia)<br>F. o. Zorkun (Ammanus, Turkey)<br>F. o. Neka (Iran)<br>F. o. Batumi / Keda (Georgia)	**1**	**34**
F. o. Shilda 1 (Georgia)<br>F. o. Bakuriani (Georgia)<br>F. o. Nichbisi (Georgia)	**2**	**3**
F. o. Akkus (Turkey)<br>F. o. Inegoel (Turkey)<br>F. s. Laghi di Monticchio (Basilicata, Italy)	**3**	**3**
F. m. Struma (Bulgaria)<br>F. s Monte Basilicò (Calabria, Italy)<br>F. s Retezat (Simeria, Romania)<br>F. o. Düzic Dumali Dag (Ammanus, Turkey)	**4**	**4**
F. o. Karabük (Turkey)	**5**	**1**
Fossil material<br>F. s Baia Mare (Romania)<br>F. o. Kondolovo (Bulgaria)	**6**	**3**

F. s Monte Pecoraro Sdirrocco (Calabria, Italy)	**7**	**1**
F. m. Borovetz (Bulgaria)<br>F. o. Asalem (Iran)	**8**	**2**
F. o. Dörtyöl (Ammanus, Turkey)	**9**	**1**
F. s Prati di Tivo (Abruzzo, Italy)<br>F. s. Medingen (Germany)<br>F. s Monte Soro Nebrodi (Sicilia, Italy)<br>F. s. Passo San Boldo (Veneto, Italy)	**10**	**4**
F. s. Alsted (Denmark)<br>F. s. Beius Bihor (Romania)<br>F. s. Des Collettes (France)<br>F. s. Annunziata (Sicilia, Italy)<br>F. s. Glarus (Switzerland)<br>F. s. Gullmarsberg (Sweden)<br>F. s. Horna Suca (Slovakia)<br>F. s. Kladska (Czech Republic)<br>F. s. Limitaciones (Spain)<br>F. s. Lowther E. (England)<br>F. s. Lozorno (Slovakia)<br>F. s. Nevrokopi (Greece)<br>F. s. Polana (Slovakia)<br>F. s. Pylion (Greece)<br>F. s. Sitno (Slovakia)<br>F. s. Slovany (Slovakia)<br>F. s. Tanap (Slovakia)<br>F. s. Tharandt (Germany)<br>F. s. Tomaszow Lubelski (Poland)<br>F. s. Vihorlat (Slovakia)<br>F. s. Westfield (Scotland)<br>F. s. Zamutov (Slovakia)<br>F. s. Zvolen (Slovakia)	**11**	**23**
F. s. Pian di Novello (Toscano, Italy)<br>F. s. Bosco della Martese (Abruzzo, Italy)	**12**	**2**

F. o. = *Fagus orientalis*; F. t. = *Fagus taurica*; F. m. = *Fagus moesiaca*; F. s. = *Fagus sylvatica*
